# Functional Insights into Silymarin as an Antiviral Agent against Enterovirus A71 (EV-A71)

**DOI:** 10.3390/ijms22168757

**Published:** 2021-08-15

**Authors:** Salima Lalani, Malihe Masomian, Chit Laa Poh

**Affiliations:** Centre for Virus and Vaccine Research, Sunway University, Subang Jaya 47500, Malaysia; salima.l@imail.sunway.edu.my (S.L.); malihem@sunway.edu.my (M.M.)

**Keywords:** enterovirus A71 (EV-A71), hand, foot and mouth disease (HFMD), flavonoids, silymarin, mechanism, resistance, mutations, antiviral

## Abstract

Enterovirus A71 (EV-A71) is a major neurovirulent agent capable of causing severe hand, foot and mouth disease (HFMD) associated with neurological complications and death. Currently, no FDA-approved antiviral is available for the treatment of EV-A71 infections. The flavonoid silymarin was shown to exert virucidal effects, but the binding site on the capsid was unknown. In this study, the ligand interacting site of silymarin was determined in silico and validated in vitro. Moreover, the potential of EV-A71 to develop resistance against silymarin was further evaluated. Molecular docking of silymarin with the capsid of EV-A71 indicated that silymarin binds to viral protein 1 (VP1) of EV-A71, specifically at the GH loop of VP1. The in vitro binding of silymarin with VP1 of EV-A71 was validated using recombinant VP1 through ELISA competitive binding assay. Continuous passaging of EV-A71 in the presence of silymarin resulted in the emergence of a mutant carrying a substitution of isoleucine by threonine (I97T) at position 97 of the BC loop of EV-A71. The mutation was speculated to overcome the inhibitory effects of silymarin. This study provides functional insights into the underlying mechanism of EV-A71 inhibition by silymarin, but warrants further in vivo evaluation before being developed as a potential therapeutic agent.

## 1. Introduction

Enterovirus A71 (EV-A71) is a highly contagious etiological agent of hand, foot and mouth disease (HFMD) which mainly infects young children (<6 years). Mild symptoms such as lesions in the mouth, hands, and feet along with fever are usually resolved within 10 days. However, hospitalization may be required to treat severe HFMD as infections by some virulent EV-A71 strains can progress to neurological stages. Currently, no antiviral therapy or vaccination has been approved clinically against HFMD by the US-FDA for the global market. Therefore, there is an urgent need to identify antivirals that can be used to treat the infection caused by neurovirulent EV-A71 strains.

Flavonoids are naturally occurring molecules with therapeutic properties. Many flavonoids have been reported to possess antiviral activities against various viruses, including Enterovirus A71 (EV-A71) [1]. Inhibition of viral entry, such as the attachment of the virus to host cells is one of the main mechanisms of action targeted by many antivirals. Baicalin was found to interfere with the human immunodeficiency virus (HIV)-1 envelope glycoprotein (gp120)-mediated fusion with human osteosarcoma cells expressing CD4/CCR5 or CD4/CXCR4 [2]. It was also reported to block the attachment of the dengue virus (DENV-2) to Vero cells by virucidal action. However, the exact binding on the dengue viral envelope was unknown [3]. Similarly, epigallocatechin gallate (EGGC) has been reported to interact with the outer surface of the herpes simplex virus (HSV) and the zika virus to impair their attachment to host cells [4]. Likewise, the influenza A virus was reported to be inhibited by flavonoids such as kaempferol, luteolin, quercetin derivatives, and tea catechins. The inhibition of the virus was mainly achieved by blocking the activity of the neuraminidase and interaction of flavonoids with hemagglutinins of the virus which inhibited the attachment of the virus to the host cells and impeded the virus entry [5,6,7,8,9].

Inhibitors preventing the attachment of the virus to host cells could do so by binding to viral structural proteins to confer inhibitory activity against EV-A71. The structural proteins of EV-A71 exposed on the surface include VP1, VP2, and VP3 while VP4 is internalized and only expelled during the uncoating stage [10]. Previously, small molecule capsid inhibitors have been reported against EV-A71, such as pleconaril [11], BTA-798 [12], and MADAL385 [13]. Pleconaril was initially considered as a potential antiviral candidate against HFMD. However, it failed to confer inhibitory activity against some clinical isolates of EV-A71 [14]. Vapendavir (BTA-798), another capsid inhibitor, was found to rigidify the capsid and thus halted the release of RNA to cause infection in vitro [12]. MADAL385, a tryptophan dendrimer, was found to act on the five-fold axis of the capsid to block the activity of EV-A71 at picomolar concentration [13]. Some of these compounds have reached clinical trials for other picornaviruses, but to date, none has progressed further against EV-A71.

A few flavonoids are known to block the attachment of EV-A71 to its cellular receptors. Formononetin, an isoflavone, has been reported to inhibit the attachment and entry of EV-A71 into Vero cells [15]. Recently, we reported that the flavonoid silymarin could inhibit EV-A71 at an inhibitory concentration (IC_50_) of 15 μg/mL by extracellular virucidal action. The compound was found to be non-cytotoxic at up to 100 μg/mL in rhabdomyosarcoma (RD) cells, with an SI value of 10.53. It was further elucidated that silymarin exerted its antiviral effect by inhibiting virus attachment to host cells [16]. However, the exact molecular inhibitory interaction between silymarin and EV-A71 remains unknown.

In this study, we identified the mechanism by which silymarin binds to the virus and inhibits the attachment of EV-A71 to its receptor(s). We also examined the effects of passaging EV-A71 in the presence of silymarin to determine the potential of EV-A71 to mutate against silymarin.

## 2. Results

### 2.1. Silymarin Interacts with VP1 In Silico

**Figure 1:**
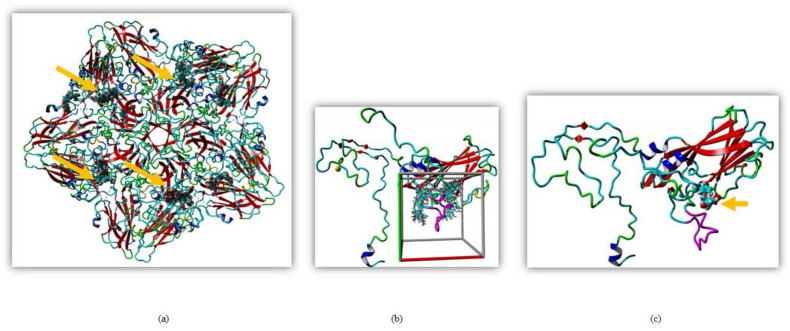
Molecular docking of silymarin with EV-A71 (**a**) Silymarin interacted with VP1 when docked on the crystal structure of EV-A71 (PDB ID: 4YVS). (**b**) Global docking of silymarin with homology modeled VP1 of EV-A71 (strain 41). The cubic cell represents specific docking near the GH loop. (**c**) Silymarin in best docking confirmation with homology modeled VP1 of EV-A71 (strain 41). The arrow indicates silymarin interacting with the GH loop (magenta).

Silymarin was previously shown to exert its antiviral effect through virucidal action but the exact mechanism was unknown [16]. To identify the interaction of the EV-A71 capsid with silymarin, global docking was performed. Since the crystal structure of EV-A71 strain 41 has not been resolved, the sequence of the capsid region of strain 41 was used to search the protein data bank Position-Specific Iterated BLAST (PSI-BLAST). The crystal structure of EV-A71 sub-genotype C4 (PDB ID: 4YVS) at a resolution of 3.65 Å showed the highest similarity (97.68%) with the capsid of EV-A71 strain 41. Therefore, 4YVS was chosen to perform global docking to identify the main target of silymarin on the capsid of EV-A71. Global docking identified that silymarin mainly interacted with the VP1 of EV-A71 (Figure 1a). 

To further elucidate the interactions between VP1 and silymarin, the structure of the VP1 of EV-A71 strain 41 was predicted by homology modeling using the YASARA software. The model was verified using pdbsum, Varify 3D, and ERRAT. Silymarin was first docked to the predicted model of VP1 which showed concentrated interactions near the GH loop of VP1 (Figure 1b). The GH loop of VP1 consists of amino acid residues from 208–222 of VP1 (YPTFGEHKQEKDLEY). Most of the residues are charged and readily available for polar interactions, with three glutamic acid and one aspartic acid residues as hydrogen acceptors and two lysines as hydrogen donor residues available for hydrogen bonding.

To identify the interaction of silymarin with amino acids of the GH loop, docking was localized near the GH loop. The amino acid residues found in close proximity with silymarin were Asp110, Ile111, Asp112, Trp203, Pro209, Glu217, Lys218, Leu220, Glu221, Gly223, Ala224, Cys225, Asn228, Met229, Met230, Phe273, Lys274, Ala275, Asn276, and Asn278 of VP1. Out of all amino acid residues in close contact, **Asp110, Ile111, Asp112, Thr210, Glu217, Lys218, Leu220, Glu221, Cys225**, and **Ans228** showed maximum binding interactions with silymarin. The best binding confirmation docked showed binding energy of 7.62 kcal/mol and the dissociation constant was determined to be 2,588,212.50 ρM (Figure 1c). 

**Figure 2:**
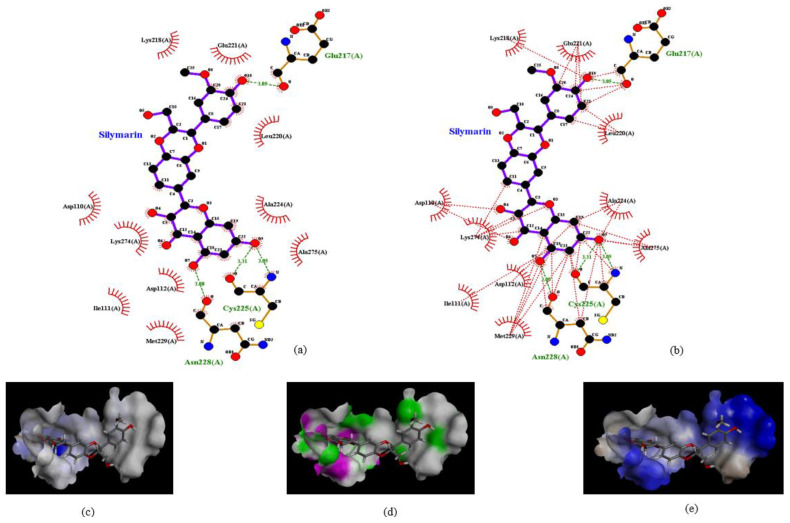
Interaction of silymarin with amino acid residues of homology modeled VP1 (EV-A71, strain 41). (**a**) The amino acids Glu217, Cys225, and Asn228 forming H-bond with silymarin and (**b**) various amino acids of VP1 interacting with silymarin with Van der Waal forces. Interactions were determined using Ligplot software (**c**). Aromatic (**d**) hydrogen (pink: donor; green: acceptor) and (**e**) hydrophobic interactions determined by Discovery studio software.

To identify the type of intermolecular bonding between these amino acid residues and silymarin, the docked structure was analyzed by the Ligplot software. Silymarin formed strong hydrogen bonds with Glu217 (H bond distance: 3.0 Å) at one end and with Ans228 (3.0 Å) and Cys225 (dual hydrogen bonds: 3.0 Å and 3.3 Å) at the other end (Figure 2a). The H-donors were contributed by amino acids of the VP1 protein, while silymarin was found to be the acceptor. Moreover, various amino acids of VP1 formed bonds with silymarin using Van der Waal forces (Figure 2b). To verify these interactions, the docked structure was further analyzed by the Discovery studio software which confirmed the interactions mapped with the Ligplot software. Despite having various aromatic rings in its structure, silymarin did not show any π-interactions with amino acids of VP1 (Figure 2c). However, amino acids involved in hydrogen bonding with silymarin and hydrophobic interactions between VP1 and silymarin could be observed in Figure 2d,e, respectively.

### 2.2. Silymarin Interacts with VP1 In Vitro

**Figure 3:**
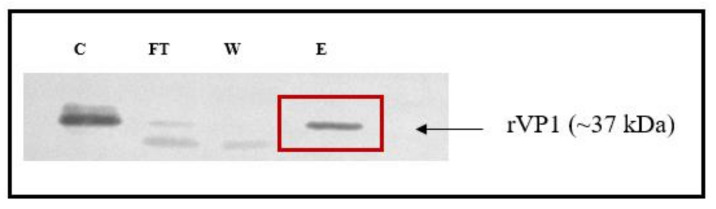
Validation of recombinant VP1 in the immunoblot. Lanes: C: crude lysate, FT: flow-through, W: wash, and E: eluate from Ni-NTA column eluted with 500 mM imidazole. A band at the 37 kDa indicated the purified recombinant VP1 protein.

To confirm and validate the interaction of silymarin with VP1, we evaluated their binding interactions in vitro using ELISA binding assay. The recombinant pRSETB-VP1 was constructed and rVP1 was expressed in *E. coli BL21*. The rVP1 was purified and the protein was confirmed by the presence of a protein band at the expected size of ~37 kDa in SDS-PAGE (Supplementary Figure S1) and Western blot (Figure 3). The actual size of VP1 is ~33 kDa; however, the expressed protein contained a 6-histidine tag (~4 kDa) and the size was found to increase to ~37 kDa. To further verify the identity of the VP1 protein, the purified protein was subjected to in-solution tryptic digestion and the extracted protein was analyzed using LC/MS. The peptide profile indicated that all the peptides matched the amino acids present in the viral protein 1 (VP1) of Enterovirus A71.

**Figure 4:**
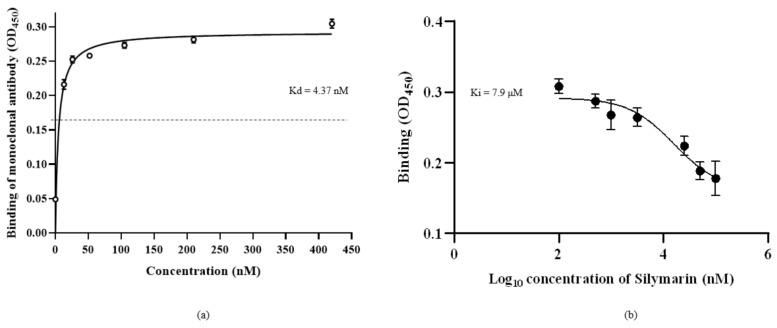
Binding of silymarin with recombinant VP1 of EV-A71. (**a**) The saturation binding curve of the anti-VP1 monoclonal antibody, clone GT185, was obtained by allowing the different concentrations of mAb to bind to rVP1 in coated ELISA plate and signals were detected using HRP conjugated secondary antibody. (**b**) The competitive binding curve of silymarin was determined by allowing different concentrations of silymarin to bind to rVP1 in the coated ELISA plate, followed by detection of rVP1 using a constant concentration (4.37 nM) of mAB as determined by the saturation binding curve. The binding constant (Kd) and inhibitory constant (Ki) were determined using nonlinear regression equations. Data represented mean values ± SEM.

The saturation binding constant (Kd) was determined by treating rVP1 with different concentrations of anti-VP1 monoclonal antibody (mAb) against rVP1 (Figure 4a). The mAb clone GT185 (GeneTex, Irvine, CA, USA) was raised against the VP1 of Enterovirus 71 BrCr strain. The Kd value was determined as 4.37 nM, indicating that the monoclonal antibody had specificity for rVP1 and could compete with silymarin to bind to rVP1. The competitive binding affinity was determined using non-linear regression analysis of inhibitor constant (Ki). Silymarin was allowed to interact with rVP1 before incubation with the anti-VP1 monoclonal antibody. It was expected that if silymarin was to bind to the rVP1, there would be reduced or no interaction between rVP1 and anti-VP1 monoclonal antibody and thus would show reduced absorbance value as compared to the control. Figure 4b indicates that silymarin as a ligand was able to successfully bind to rVP1, preventing the interaction between rVP1 and anti-VP1 antibody, and thus contributed to the lower absorbance observed. The logKi value determined was log 3.9 nM (7.9 µM). 

### 2.3. Genetic Stability of EV-A71 in the Presence of Silymarin

**Figure 5:**
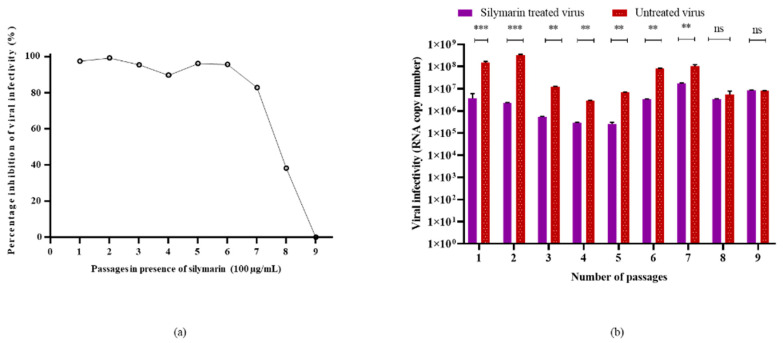
Susceptibility of EV-A71 to silymarin over 9 continuous passages. (**a**) Inhibition of EV-A71 in the presence of 100 µg/mL silymarin over nine passages. (**b**) Virus titers of EV-A71 passaged in the presence of 100 µg/mL silymarin (S1–S9) and titers of EV-A71 passaged without silymarin in media (V1–V9) in media. ** *p* < 0.01, *** *p* < 0.001, indicate a significant difference analyzed by *t*-test. ns = non-significant.

To evaluate the genetic stability of EV-A71 in the presence of silymarin, the virus was passaged in the presence and absence of silymarin for nine continuous passages. Viral RNA was extracted from the tissue culture supernatant from each passage and the viruses were subjected to qRT-PCR analysis for the determination of infectivity (RNA copy number). Figure 5 shows the inhibition of EV-A71 by 100 µg/mL silymarin. Growth of EV-A71 was susceptible to inhibition by 100 µg/mL silymarin until 7 passages with more than 80% of inhibition. However, the virus started to develop resistance at passage number 8, indicated by a reduction in inhibition and viral titers followed by complete resistance to inhibition at passage 9 (Figure 5). The cytopathic effects (CPE) in treated and untreated cells at passage 9 were observed to be the same and therefore, no inhibition was evident (Supplementary Figure S2).

### 2.4. Identification of Resistant Mutant

**Figure 6:**
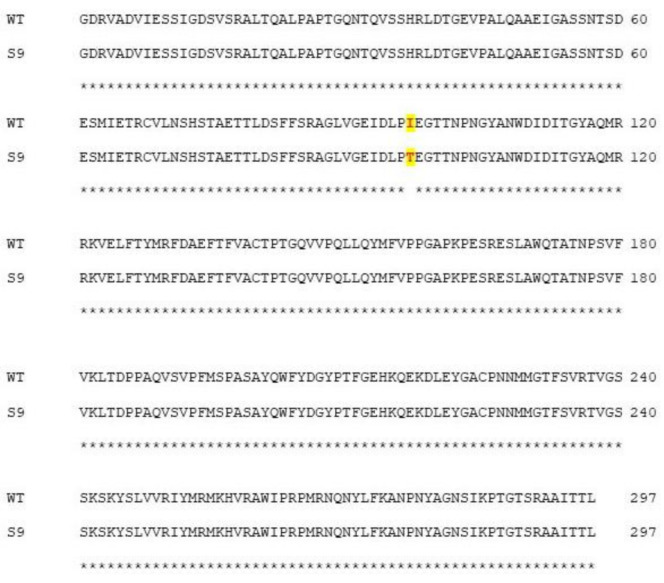
Sequence alignment analysis of wild type (WT) and silymarin-resistant mutant (S9) by Clustal Omega 1.2.4. The amino acid mutation from isoleucine (I) in wild type to threonine (T) in the silymarin-resistant mutant is highlighted.

To confirm the observation of CPE and reduced viral titers from in vitro assays, Sanger sequencing of wild type (WT), silymarin-resistant mutant (S9), and EV-A71 passaged in the absence of silymarin at passage 9 (V9) was carried out. A single point mutation that resulted in the substitution of the amino acid isoleucine (I) by threonine (T) at position 97 in the BC loop of VP1 protein was found to be associated with resistance of EV-A71 to the flavonoid silymarin (Figure 6). Besides the I97T substitution in VP1, there were no differences found in the genome of the S9 mutant when it was compared to the wild type. The sequencing data confirmed that the mutation arose due to the selection pressure exerted by silymarin in the culture.

### 2.5. Structural Analysis of Mutation

**Figure 7:**
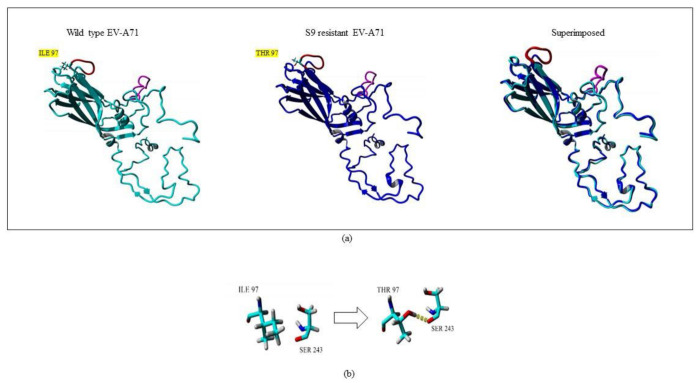
Superposition of wild type (WT) and silymarin-resistant mutant (S9) by YASARA v2020. (**a**) The cyan and blue structures represent WT and S9, respectively. The GH loop is shown in magenta while the BC loop is shown in red color. The amino acid mutation from isoleucine (I) in wild type to threonine (T) in the silymarin-resistant mutant in BC loop is highlighted in yellow. (**b**) A close-up view of the point mutation from amino acid isoleucine (I) in wild type to threonine (T) in the silymarin-resistant mutant (S9). The hydrogen bond observed is indicated as yellow dotted lines.

Amino acid changes in structural proteins could greatly influence the stability and folding of a protein. These changes could also contribute to overall changes in the hydrogen bonding amongst the amino acids and subsequently enhance or reduce the rigidity of the structure. To understand the impact that the mutation I97T had on VP1, the structure of S9 was modeled. The 3D structure of S9 was superimposed with the structure of WT VP1. Interestingly, there was no obvious conformational change in the whole structure of S9 when compared to wild type virus. The structures had only 0.68 Å root mean square deviation (RMSD) over 296 matched carbon-α of their amino acids. However, the mutation I97T was observed to give rise to an additional hydrogen bond which was absent in the wild type VP1 structure. The amino acid threonine carries an additional hydroxyl group which is available for hydrogen bonding. The presence of T at position 97 gave rise to an additional hydrogen bond with S243 (Figure 7). Serine at position 243 is crucial as it is present adjacent to the positively charged K242 and K244 residues in the five-fold axis of VP1 which could stabilize its conformation. This stability could confer effective binding of the virus to the host receptors.

### 2.6. In Silico Binding of Silymarin to S9 Mutant

**Figure 8:**
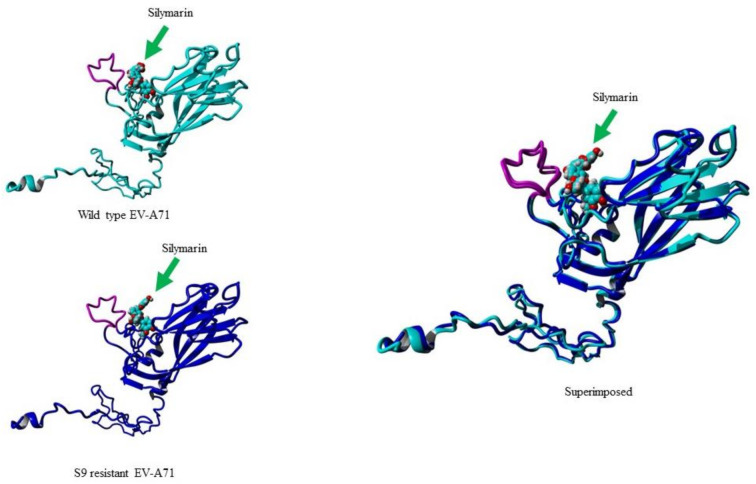
Molecular docking of silymarin with wild type (WT) and silymarin-resistant mutant (S9) of EV-A71 by YASARA v2020. Silymarin in best docking confirmation with homology modeled VP1 of wild type (cyan) and S9 mutant virus (blue). The arrow indicates silymarin interacting with the GH loop (magenta) at the similar position.

When the structure of the VP1 of the S9 mutant was compared to the structure of wild type virus VP1, the structures had only 0.68 Å root mean square deviation (RMSD) over 296 matched carbon-α of their amino acids. To examine the binding ability of silymarin with mutated VP1, silymarin was docked to the VP1 of the S9 mutant and analyzed for comparative binding energies. The best binding confirmation docked showed a binding energy of 6.53 kcal/mol which was less than that of wild type virus (7.62 kcal/mol), suggesting the reduced binding of silymarin to the VP1 of the S9 mutant. Out of all amino acid residues in close contact, **Asp110**, **Ile111**, **Asp112**, **Pro209**, **Leu220**, **Glu221**, **Ala224**, **Ans228**, **Met229**, **Phe273**, **Ala275**, and **Asn276** showed maximum binding interactions with silymarin. Interestingly, silymarin also interacted with most of these amino acids of wild type virus, suggesting the accuracy of in silico modeling. Furthermore, it also indicated that the binding of silymarin is primarily near the GH loop (Figure 8).

### 2.7. Virus Binding Ability of the Mutant Virus (S9) to Host Cells

**Figure 9:**
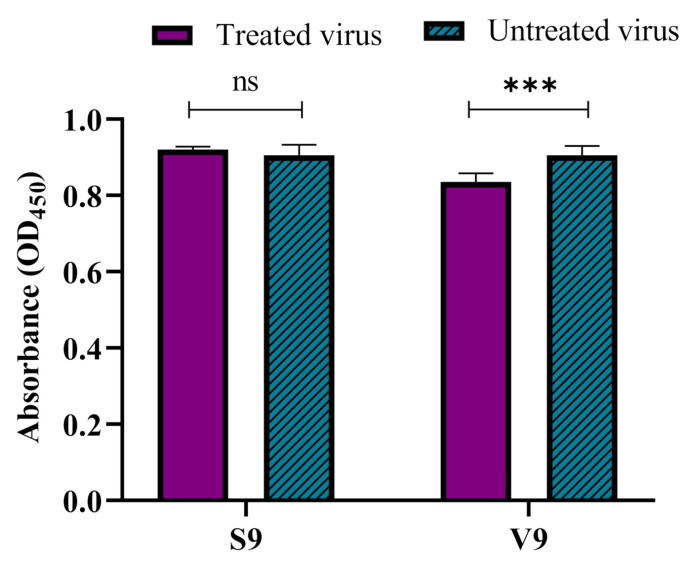
The increased binding ability of the S9 mutant virus to RD cells. The increase in the binding ability of the S9 mutant virus was determined by a cell-based competitive ELISA assay. The V9 virus (wild-type virus passaged in the absence of silymarin) was used as a control. The silymarin treated or untreated S9 mutant and V9 viruses were allowed to bind to RD cells, followed by detection of the bound virus using a monoclonal antibody against VP1 of EV-A71 (Genetex, clone GT185). Data represented as mean values ± SEM of at least two independent experiments and six replicates. *** *p* < 0.001, indicates a significant difference analyzed by *t*-test; ns = non-significant.

Sanger sequence analysis revealed that the single mutation in the VP1 gene of mutant virus (S9) gave rise to a I97T substitution in VP1. To determine the impact of the mutation on the binding of the mutant virus, an ELISA-based virus binding assay was performed. The results demonstrated that the binding ability of the mutant virus (S9) was significantly increased and there were no significant differences between the binding ability of the silymarin-treated S9 and untreated S9 virus to the RD cells. In comparison, the V9 virus (wild-type virus after 9 passages in absence of silymarin) was also evaluated to verify the binding ability of the V9 virus. The results showed that there was a significant difference between the silymarin treated and the untreated V9 virus (Figure 9). Thus, the results from the virus binding assay confirmed that mutation I97T harbored by the S9 mutant in the BC loop of VP1 increased the binding of the virus to the host cells.

## 3. Discussion

Enterovirus A71 (EV-A71) is a neurotropic viral pathogen of rising concern for pediatric health in the Asia Pacific region. Some neurovirulent strains can cause neurological symptoms such as encephalitis, meningitis, acute respiratory distress, and pulmonary edema which can lead to high fatality. To date, no FDA-approved therapeutic antiviral drugs are available against EV-A71. Plant-derived flavonoids have been reported to inhibit EV-A71 replication in cultures by targeting post-attachment stages such as polyprotein processing or preventing EV-A71 replication by interfering with IRES activity via FUBP and hNRP proteins [17,18].

Recently, we reported that the flavonoid silymarin could inhibit EV-A71 by a virucidal mode of action by interacting with the virus blocking its attachment to host cells. However, the exact mechanism of action was unknown [16]. Since we previously reported that pre-treatment of RD cells with silymarin and post-infection did not show significant inhibition of EV-A71, the binding site of silymarin was probably with the surface of the viral particle. In this study, we determined that silymarin interacted with the highly surface-exposed viral protein VP1. The region of VP1 involved in the binding was identified by molecular docking as the GH loop of EV-A71.

The GH loop has great importance in the mechanism of viral entry and interaction of the virus with host cells. The GH loop of VP1 spans from amino acid residues 208 to 222 and plays a crucial role during the attachment and uncoating of the virus. The GH loop was reported to interact with the uncoating receptor SCARB2 to form an uncoating complex and this interaction allows the release of the pocket factor, followed by a pocket collapse that permits the genomic RNA to be released into the cells [19]. If silymarin interacts with amino acids Thr210, Glu217, Lys218, Leu220, and Glu221 present in the GH loop of VP1 as suggested by molecular docking, this could explain the mode of virucidal action exerted by silymarin. Furthermore, the adaptor–sensor mechanism of EV-A71 involves amino acid residues 230–233 in the VP1 of EV-A71. Interestingly, the amino acids preceding the residues involved in the adaptor–sensor mechanism of the virus were also found to interact with silymarin by hydrogen bonds (Cys225 and Ans228) and Van der Waal forces (Ala224 and Met229). These interactions might have contributed to the disruption of the uncoating process, resulting in inhibition of EV-A71.

Silymarin contains multiple hydroxyl groups that could form hydrogen bonds with amino acids in VP1. Not many molecules are known to interact with the GH loop of VP1 to exert their antiviral effects, thus making silymarin a unique antiviral molecule that interacted with amino acid residues in the GH loop which led to the inhibition of the attachment of EV-A71 virions. Previously, neutralizing monoclonal antibodies (D5, H7, C4) were reported to identify the conserved epitopes on the GH loop that could inhibit the attachment, entry, and uncoating of the virus in the tissue culture. The amino acids of the GH loop critical for the interaction with antibodies were mapped as residues 215 to 219 [20]. Notably, the same region has been reported as the site of interaction of SCARB2 with the capsid of EV-A71. The interaction of any ligand (small molecule or antibody) at the GH loop of VP1 would cause steric hindrance for SCARB2 to bind to the residues of VP2 and VP3 required for receptor attachment [21]. Interestingly, silymarin was also found to interact with amino acids 217, 218, and the residues downstream of the GH loop (222–229) by molecular docking, possibly causing interference in receptor binding.

To substantiate that silymarin interacted with VP1 of EV-A71 as elucidated by molecular docking, the binding of silymarin with recombinant VP1 was evaluated in vitro. It was found that increasing concentrations of silymarin were able to inhibit the binding of monoclonal antibody (GT185) to VP1 of EV-A71 in a competitive manner. The finding inferred that either silymarin or the antibody had the same binding site or binding of silymarin to VP1 made the binding site inaccessible to the antibody. In either case, the binding of silymarin with VP1 predicted in the in silico study was validated.

The region around position 97 in the BC loop of the VP1 of EV-A71 has been reported as a hot spot for mutations. The BC loop of VP1 is involved in the binding of the EV-A71 to the host receptors and any change in amino acids of the BC loop could greatly influence receptor binding. Cordey et al. (2012) reported that the change of amino acid from the hydrophobic leucine residue to the charged residue arginine at position 97 increased neural cell tropism in neuroblastoma cells [22]. The mutation L97R was also found in a patient with a severe neurological case of meningitis, suggesting a replicative advantage of the L97R-mutant virus in the CNS [23]. Double mutations resulting in glutamine to glutamic acid replacements at positions 98 and 145 (Q98E and Q145E) were reported to completely abolish the binding of the virus to the heparin sulphate receptor. However, compensatory mutations (Q98K or E145Q/G) were soon acquired by the mutated virus to restore binding to the heparin sulphate receptor [24].

EV-A71 has been shown to confer resistance against capsid binding inhibitors by carrying a mutation at position 97 or 98 in the BC loop. A suramin derivative NF449 known to inhibit the attachment of EV-A71 to heparin sulphate and PSGL-1 in RD cells was found to lose its susceptibility when the amino acids in VP1 at positions 98 and 244 were changed from glutamic acid to glutamine (E98Q) and lysine to arginine (K244R), respectively. Both these mutations caused a conformational change in the structure of the five-fold axis, favoring attachment of the virus with the host receptor and hence resulted in the resistance to the NF449 inhibitor [25].

In this study, genome analysis of the resistant mutant identified a substitution of isoleucine by threonine (I97T) at position 97 of the BC loop. Interestingly, the mutation on the genome leading to I97T has previously been identified by Tan et al. (2017) to significantly increase the binding of the mutant virus to the heparin sulphate receptor [24]. The results obtained through virus binding assays confirmed the earlier report of Tan et al. indicating that the I97T mutation led to increased binding of the mutant virus to the host cell. The substitution I97T also resulted in an additional hydrogen bond with serine at position 243 of VP1. S243 plays a crucial role as it is present in adjacent position to the positively charged K242 and K244 residues in the five-fold axis of VP1, which could stabilize its conformation. This stability could confer effective binding of the virus to the host receptors. Remarkably, S243 was found to be involved in SCARB2-independent uncoating of EV-A71 using the cyclophilin A receptor [26].

Strong binding of the S9-mutant virus to heparan sulphate receptor could have permitted attachment to the host cells followed by entry of the virus using the alternative uncoating receptor cyclophilin A to over-ride the inhibition by silymarin at the GH loop. The increased binding ability of the virus and availability of secondary uncoating receptor (cyclophilin A) in the near vicinity could provide an explanation for the emergence of the mutant virus resistant to silymarin. It also suggests that although silymarin interacted with the virus at the GH loop, the virus could induce a compensatory mutation far from the ligand interacting site in the BC loop of VP1.

Nevertheless, to further validate the in silico observation that silymarin was binding to the GH loop, there is a need to conduct in vitro experiments using the GH loop specific peptide or monoclonal antibody, such as D5, that could bind to the GH loop and serve as a strong competitive inhibitor against silymarin in competitive ELISA assays with rVP1. Moreover, amino acids which were found to be involved in the binding of silymarin to the GH loop in silico could be altered individually by performing site-directed mutagenesis studies to identify the molecular interaction of silymarin with the VP1 of EV-A71.

In conclusion, this study identified that silymarin inhibited EV-A71 by interaction with VP1 to disrupt viral attachment and uncoating. Silymarin had the added advantage of exerting extracellular virucidal action and therefore, it is more bioavailable for antiviral activity. Silymarin was shown to interact with the GH loop of VP1 of EV-A71 by molecular docking analysis. Interaction with VP1 was validated in competitive ELISA with monoclonal antibody GT185 which was specific for VP1 of EV-A71. The binding affinity of silymarin for VP1 could further be improved by the addition of functional groups at interacting positions that could confer stronger binding with EV-A71. The in silico results that indicated the binding of silymarin to the GH loop of VP1 could be further studied by the GH loop specific antibody or peptide that may serve as a competitive inhibitor to silymarin. An in-depth understanding of the molecular mechanism using site-directed mutagenesis could also be employed in future studies. Moreover, silymarin might act synergistically with other flavonoids such as prunin or isorhamnetin that have been shown to exhibit different mechanisms of inhibition. The potential of silymarin to prevent mice from lethal challenge could be further explored before recommending it to be used as a potential antiviral against EV-A71 clinically.

## 4. Materials and Methods

### 4.1. Cells and Virus

Rhabdomyosarcoma (RD, CCL-136, ATCC, Manassas, VA, USA) cells were maintained with Dulbecco’s Modified Eagle’s Medium (DMEM) supplemented with 10% fetal bovine serum (FBS) and 1% penicillin–streptomycin antibiotics (PSA). Enterovirus A71 (EV-A71) strain 41 (5865/SIN/000009 strain, Genebank accession no. AF316321.2) was propagated in RD cells. 

### 4.2. In Silico Studies

#### 4.2.1. Three-Dimensional Structure Prediction of VP1 of EV-A71

The PSI-BLAST (Position-Specific Iterated BLAST) of the National Centre for Biotechnology Information (http://www.ncbi.nlm.nih.gov/BLAST accessed on 6 July 2020 and 15 June 2021) was conducted to search for a suitable crystal structure in the protein structure database with more than 95% identity to use as a template. The 3D structure of VP1 of EV-A71 virus sub-genotype B4 strain 41(5865/SIN/000009) was predicted by homology modeling through the YASARA structure (v2020) using the chain ‘A’ of 3D structures of 3ZFE, 6Z3P, 4CEW, 4CDQ, and 3VBH as templates.

An unrestrained high-resolution refinement with explicit solvent molecules was run using YASARA 03 force fields. The result was validated by the YASARA program to ensure that the refinement did not move the model in the wrong direction. Based on the templates used to predict the structure, 7 models were built. To refine the geometry and orientation of the binding model, the highest-ranked models for each template were subsequently refined using the YASARA program. YASARA combined the best parts of the 7 models to obtain a hybrid model with the aim to increase the accuracy beyond each of the contributors. The resulting hybrid model obtained with the highest quality *Z*-scores was saved as the final model. Subsequently, the predicted model was evaluated using PROCHECK (http://www.ebi.ac.uk/thornton-srv/software/PROCHECK/ accessed on 7 July 2020 and 16 June 2021), Verify 3D (https://servicesn.mbi.ucla.edu/Verify3d/ accessed on 7 July 2020 and 16 June 2021), and ERRAT (https://servicesn.mbi.ucla.edu/ERRAT/ accessed on 7 July 2020 and 16 June 2021) programs.

#### 4.2.2. Molecular Docking Study

An in silico study was used to predict and define the binding location of silymarin to the capsid protein of EV-A71. The 3D structure of silymarin was retrieved from PubChem (Chemical ID: 1548994). Based on PSI-BLAST results, the closest whole capsid protein structure to EV-A71 virus sub-genotype B4 strain 41 was EV-A71 subgenotype C4 with PDB ID 4YVS that was used for global docking. Based on the docking result of whole capsid proteins with silymarin, in the next round, silymarin was docked to the structure of VP1 where the GH loop was identified as interacting site. To fit the GH loop and allow nonconstructive binding, the grid box was set to cover the amino acids 208 to 222 of VP1. Using the YASARA docking program, binding energy and dissociation constant (Kd) were extracted from atomic B-factor and atomic property. More positive energies indicated stronger binding, and negative energies meant weaker or no binding. For each round of docking, 35 runs were performed, and the docking results cluster around certain hotspot conformations. The lowest energy complex in each cluster was saved. The final docked structure was analyzed by the Ligplot (EMBL-EBI, Cambridgeshire, UK) and Discovery studio (Dassault Systèmes, Vélizy-Villacoublay, France) software. A similar method was used for docking of silymarin to the VP1 of the S9 mutant virus.

### 4.3. Construction of Plasmid with Recombinant VP1

The pRSETB plasmid (Invitrogen™, Life Technologies, Waltham, MA, USA) and *E. coli BL21* (DE3) strain were kind gifts from Associate Professor Sharifah Syed Hassan, Monash University, Malaysia. The plasmid containing the VP1 gene (pRSETB-VP1) of EV-A71 was constructed by GenScript (Piscataway, NJ, USA). The recombinant pRSETB-VP1 was sequenced to identify any nonsense or missense mutations. To express the VP1 protein, a single colony of *E. coli BL21* containing pRSETB-VP1 was grown overnight in 10 mL Luria–Bertani (LB) broth supplemented with 100 µg/mL ampicillin in a shaking incubator (37 °C, 200 rpm). An aliquot (2 mL) of the overnight culture was inoculated into in 200 mL LB broth and grown in a shaking incubator (37 °C, 200 rpm) until the OD_600_ reached ~0.5. The culture was subjected to IPTG induction (0.1 M) for 24 h at 37 °C. The culture was harvested by centrifugation at 10,000× *g* for 10 min at 4 °C. Cells were sonicated (30 s ON/30 s OFF, 4 min) and proteins were cleared by centrifugation at 10,000× *g* for 10 min at 4 °C. The supernatant was used for protein purification.

### 4.4. Protein Purification

Protein purification was performed using the HisPur Ni-NTA Resin Kit (Thermo Fisher Scientific, Life Technologies, Waltham, MA, USA) according to the manufacturer’s instructions with minor modifications. Briefly, the column was washed with 5 column volumes (CVs) of distilled water. The column was flushed with 5 CVs equilibrium buffer (Tris buffer pH 8.3) containing 5 mM imidazole. The proteins were allowed to pass through the Ni-NTA column and flow-through was collected. The column was washed with a wash buffer (Tris buffer, pH 8.3) containing 10 mM imidazole until the OD of the flow-through reached ~0.01. Proteins were eluted using an elution buffer containing 500 mM imidazole. The proteins in the crude extract, flow-through sample, washes, and eluates were separated in a 12% SDS PAGE which was electrophoresed for 120 min at 100 V. To verify the identity of the rVP1 protein, the separated proteins were analyzed by western blotting. Moreover, purified protein from final eluate was subjected to in-solution tryptic digestion for LC/MS analysis.

### 4.5. ELISA Binding Assay

ELISA binding assay was performed as described by Ahmad et al. (2017) with minor modifications [27]. First, a saturation binding assay was performed to determine the specificity of the anti-VP1 monoclonal antibody, clone GT185 (GeneTex, Irvine, CA, USA) with the recombinant VP1 (rVP1). Briefly, an ELISA plate was coated with purified recombinant VP1 (rVP1) diluted in Tris-HCl buffer (pH 8.3) overnight at 4 °C. The following day, the plate was washed with Tris-buffered saline (TBS) containing 0.05% Tween 20 (TSBST) and blocked with 1% bovine serum albumin (BSA) in TBS (BTBS) for 3 h at room temperature. Serially diluted concentrations of anti-VP1 monoclonal antibody in TBST containing 1% BSA (BTBST) were incubated with rVP1 for 2 h at room temperature. After incubation, the plate was washed twice with TBST and a goat anti-mouse IgG HRP secondary antibody (Santa Cruz Biotechnology, Dallas, TX, USA) was added and incubated for 1 h at room temperature. The excess antibody was removed by washing the plate with TBST. The substrate 3,3′,5,5′-Tetramethylbenzidine (TMB) (SeraCare Life Sciences Inc, Milford, MA, USA) was added and the plate was incubated in the dark for 10 min. A stop solution of 1 M sulphuric acid (100 µL) was added and binding of the antibody with rVP1 was measured at 450 nm using Infinite 200 Pro Multiplate Reader (Tecan, Männdedorf, Switzerland).

For the competitive binding assay, ELISA plate was coated with the purified recombinant VP1 (rVP1) diluted in Tris-HCl buffer overnight at 4 °C. The following day, the plate was washed twice with TBST and blocked with 1% BTBS for 3 h at room temperature. Various concentrations of silymarin (100, 50, 25, 10, 1, 0.5, 0.1 µM) were prepared in 1% BTBST and incubated with rVP1 for 2 h at room temperature. Silymarin was removed and the plate was washed twice with TSBT, followed by 2 h incubation with the anti-VP1 primary antibody at the concentration of 4.37 nM. After incubation, the plate was washed twice with TBST and goat anti-mouse IgG HRP secondary antibody (Santa Cruz Biotechnology, Dallas, TX, USA) was added for 1 h incubation at room temperature. The excess antibody was removed by washing the plate with TBST. The substrate 3,3′,5,5′-Tetramethylbenzidine (TMB) (SeraCare Life Sciences Inc, Milford, MA, USA) was added and the plate was incubated in the dark for 10 min. A stop solution of 1 M sulphuric acid (100 µL) was added and binding of the antibody with rVP1 was measured at 450 nm using Infinite 200 Pro Multiplate Reader (Tecan, Männdedorf, Switzerland). The binding curve was constructed and the inhibitory constant (Ki) of silymarin was determined using GraphPad Prism software v7 (GraphPad Software, San Diego, CA, USA).

### 4.6. Isolation of Resistant Mutant(s) in the Presence of Silymarin

Isolation of mutant resistant to silymarin was conducted as described by Qing et al. (2014) with modifications [26]. Briefly, RD cells (3 × 10^5^) grown in 6-well plates were infected with 100 µg/mL silymarin-treated EV-A71 (MOI = 0.1) for 1 h. Simultaneously, wild-type EV-A71 was also used to infect RD cells at MOI = 0.1 for 1 h as control. The inocula were removed and RD cells were allowed to grow in the presence or absence of 100 µg/mL silymarin. The virus present in the supernatant was collected after appearance of cytopathic effects (CPE) or after 24 h. The supernatant collected was centrifuged at 4000× *g* for 5 min at 4 °C to remove cell debris. The cleared supernatant was subsequently used at MOI 0.1 for infection of a new monolayer of RD cells (passage 1). The passage was repeated 9 times using the same method described above for passage 1 until the appearance of the resistant phenotype as indicated by CPE in RD cells. Virus passaged in the absence of silymarin was used as a control to validate the mutations caused by serial passaging in the presence of silymarin. All the supernatants containing viruses were kept at −80 °C until RNA was extracted from viruses for qRT-PCR and genome sequencing.

### 4.7. RNA Extraction and Real-Time Quantitative Reverse Transcriptase-Polymerase Chain Reaction (qRT-PCR)

The RNA from the tissue culture supernatants containing viruses was extracted using the QIAamp ViralRNA mini kit (Qiagen, Hilden, Germany) according to the manufacturer’s instructions. TaqMan^®^ Fast virus 1-step master mix (ABI, Carlsbad, CA, USA) was used to carry out qRT-PCR in the CFX96 TouchTM RealTime PCR Detection System (Bio-Rad, Hercules, CA, USA) as described by Lalani et al. (2020) [16].

### 4.8. cDNA Preparation and Genome Sequencing

The cDNA was prepared from RNA extracted for genome sequencing using SuperScriptTM IV First Strand Synthesis System (ThermoFisher Scientific, Waltham, MA, USA), following the manufacturer’s protocol using a gene-specific reverse primer (3D-3′UTR: GCTATTCCGGTTATAACAAATTTACC). The cDNA was amplified with Q5^®^ High-Fidelity DNA Polymerase polymerase (NEB, Ipswich, MA, USA) using the 5′UTR forward (TTAAAACAGCTGTGGGTTGTAC) and 3D-3′UTR reverse (GCTATTCCGGTTATAACAAATTTACC) primers. The PCR product was run in a 1% agarose gel to confirm the presence of the genomic DNA of EV-A71. The PCR product was extracted and sequenced. The data was analyzed using Geneious Software (Biomatters, Auckland, New Zealand) and Clustal Omega (Conway Institute UCD, Dublin, Ireland).

### 4.9. Virus Binding Assay

To determine the increased binding ability of mutant virus passaged in the presence of silymarin as compared to virus passaged without treatment, a virus binding assay (cell-based ELISA) was performed according to Spurgers et al. (2013), with minor modifications [28]. RD cells (2 × 10^5^/mL) were grown overnight in the wells of a 96-well plate. The next day, mutant virus (S9) and untreated wild-type virus (V9) were subjected to treatment with 100 μg/mL silymarin for 1 h at 37 °C, followed by 5 min incubation at 4 °C. Pre-chilled RD cells were infected with the pre-chilled silymarin-treated viruses (S9 and V9) and incubated at 4 °C for 1 h to allow virus binding. The inoculum was removed after 1 h and unbound viruses were washed away with ice-cold PBS. RD cells were fixed with 80% acetone for 30 min, followed by 1 h incubation with the anti-VP1 primary monoclonal antibody (1:2000) in 1% BSA at room temperature. After incubation, the 96-well plate was washed with TBST and 1:2000 diluted goat anti-mouse IgG HRP secondary antibody (Santa Cruz Biotechnology, Dallas, TX, USA) was added and incubated for 1 h at room temperature. The excess antibody was removed by washing the plate with TBST. The substrate 3,3′,5,5′-Tetramethylbenzidine (TMB) (SeraCare Life Sciences Inc, Milford, MA, USA) was added and the plate was incubated in the dark for at least 20 min. A stop solution of 1 M sulphuric acid (100 µL) was added and binding of the antibody with S9 or V9 viruses was measured at 450 nm using Infinite 200 Pro Multiplate Reader (Tecan, Männdedorf, Switzerland).

### 4.10. Statistical Data Analysis

The data were analyzed using GraphPad Prism, v7 (GraphPad Software, San Diego, CA, USA) to determine the binding constant (Kd) and inhibitory constant (Ki) values. Data presented are the average ± S.E.M of three independent experiments and analyzed by student *t*-test (* *p* < 0.05, ** *p* < 0.01, *** *p* < 0.001).

## Data Availability

The datasets used and/or analyzed during the current study are available from the corresponding author on reasonable request.

## Supplementary Materials

The following are available online at https://www.mdpi.com/article/10.3390/ijms22168757/s1.

## References

[1] Lalani S., Poh C.L. (2020). Flavonoids as Antiviral Agents for Enterovirus A71 (EV-A71). Viruses.

[2] Li B.Q., Fu T., Dongyan Y., Mikovits J.A., Ruscetti F.W., Wang J.M. (2000). Flavonoid baicalin inhibits HIV-1 infection at the level of viral entry. Biochem. Biophys. Res. Commun..

[3] Moghaddam E., Teoh B.-T., Sam S.-S., Lani R., Hassandarvish P., Chik Z., Yueh A., Abubakar S., Zandi K. (2014). Baicalin, a metabolite of baicalein with antiviral activity against dengue virus. Sci. Rep..

[4] Carneiro B.M., Batista M.N., Braga A.C.S., Nogueira M.L., Rahal P. (2016). The green tea molecule EGCG inhibits Zika virus entry. Virology.

[5] Jeong H.J., Ryu Y.B., Park S.-J., Kim J.H., Kwon H.-J., Kim J.H., Park K.H., Rho M.-C., Lee W.S. (2009). Neuraminidase inhibitory activities of flavonols isolated from Rhodiola rosea roots and their in vitro anti-influenza viral activities. Bioorg. Med. Chem..

[6] Yi L., Li Z., Yuan K., Qu X., Chen J., Wang G., Zhang H., Luo H., Zhu L., Jiang P. (2004). Small molecules blocking the entry of severe acute respiratory syndrome coronavirus into host cells. J. Virol..

[7] Wu W., Li R., Li X., He J., Jiang S., Liu S., Yang J. (2016). Quercetin as an antiviral agent inhibits influenza A virus (IAV) entry. Viruses.

[8] Tao J., Hu Q., Yang J., Li R., Li X., Lu C., Chen C., Wang L., Shattock R., Ben K. (2007). In vitro anti-HIV and -HSV activity and safety of sodium rutin sulfate as a microbicide candidate. Antivir. Res..

[9] Roschek B., Fink R.C., McMichael M.D., Li D., Alberte R.S. (2009). Elderberry flavonoids bind to and prevent H1N1 infection in vitro. Phytochemistry.

[10] Yuan J., Shen L., Wu J., Zou X., Gu J., Chen J., Mao L. (2018). Enterovirus A71 Proteins: Structure and Function. Front. Microbiol..

[11] Shia K.-S., Li W.-T., Chang C.-M., Hsu M.-C., Chern J.-H., Leong M.K., Tseng S.-N., Lee C.-C., Lee Y.-C., Chen S.-J. (2002). Design, Synthesis, and Structure−Activity Relationship of Pyridyl Imidazolidinones: A Novel Class of Potent and Selective Human Enterovirus 71 Inhibitors. J. Med. Chem..

[12] Tijsma A., Franco D., Tucker S., Hilgenfeld R., Froeyen M., Leyssen P., Neyts J. (2014). The Capsid Binder Vapendavir and the Novel Protease Inhibitor SG85 Inhibit Enterovirus 71 Replication. Antimicrob. Agents Chemother..

[13] Sun L., Lee H., Thibaut H.J., Lanko K., Rivero-Buceta E., Bator C., Martinez-Gualda B., Dallmeier K., Delang L., Leyssen P. (2019). Viral engagement with host receptors blocked by a novel class of tryptophan dendrimers that targets the 5-fold-axis of the enterovirus-A71 capsid. PLoS Pathog..

[14] Shih S.-R., Tsai M.-C., Tseng S.-N., Won K.-F., Shia K.-S., Li W.-T., Chern J.-H., Chen G.-W., Lee C.-C., Lee Y.-C. (2004). Mutation in Enterovirus 71 Capsid Protein VP1 Confers Resistance to the Inhibitory Effects of Pyridyl Imidazolidinone. Antimicrob. Agents Chemother..

[15] Li G., Gao Q., Yuan S., Wang L., Altmeyer R., Lan K., Yin F., Zou G. (2017). Characterization of three small molecule inhibitors of enterovirus 71 identified from screening of a library of natural products. Antivir. Res..

[16] Lalani S.S., Anasir M.I., Poh C.L. (2020). Antiviral activity of silymarin in comparison with baicalein against EV-A71. BMC Complement. Med. Ther..

[17] Gunaseelan S., Wong K.Z., Min N., Sun J., Ismail N.K.B.M., Tan Y.J., Lee R.C.H., Chu J.J.H. (2019). Prunin suppresses viral IRES activity and is a potential candidate for treating enterovirus A71 infection. Sci. Transl. Med..

[18] Lv X., Qiu M., Chen D., Zheng N., Jin Y., Wu Z. (2014). Apigenin inhibits enterovirus 71 replication through suppressing viral IRES activity and modulating cellular JNK pathway. Antivir. Res..

[19] Wang X., Peng W., Ren J., Hu Z., Xu J., Lou Z., Li X., Yin W., Shen X., Porta C. (2012). A sensor-adaptor mechanism for enterovirus uncoating from structures of EV71. Nat. Struct. Mol. Biol..

[20] Ku Z., Ye X., Shi J., Wang X., Liu Q., Huang Z. (2015). Single Neutralizing Monoclonal Antibodies Targeting the VP1 GH Loop of Enterovirus 71 Inhibit both Virus Attachment and Internalization during Viral Entry. J. Virol..

[21] Dang M., Wang X., Wang Q., Wang Y., Lin J., Sun Y., Li X., Zhang L., Lou Z., Wang J. (2014). Molecular mechanism of SCARB2-mediated attachment and uncoating of EV71. Protein Cell.

[22] Cordey S., Petty T.J., Schibler M., Martinez Y., Gerlach D., Van Belle S., Turin L., Zdobnov E., Kaiser L., Tapparel C. (2012). Identification of Site-Specific Adaptations Conferring Increased Neural Cell Tropism during Human Enterovirus 71 Infection. PLOS Pathog..

[23] Brown B.A., Oberste M.S., Alexander J.P., Kennett M.L., Pallansch M.A. (1999). Molecular Epidemiology and Evolution of Enterovirus 71 Strains Isolated from 1970 to 1998. J. Virol..

[24] Tan C.W., Sam I.C., Lee V.S., Wong H.V., Chan Y.F. (2017). VP1 residues around the five-fold axis of enterovirus A71 mediate heparan sulfate interaction. Virology.

[25] Arita M., Wakita T., Shimizu H. (2008). Characterization of pharmacologically active compounds that inhibit poliovirus and enterovirus 71 infectivity. J. Gen. Virol..

[26] Qing J., Wang Y., Sun Y., Huang J., Yan W., Wang J., Su D., Ni C., Li J., Rao Z. (2014). Cyclophilin A Associates with Enterovirus-71 Virus Capsid and Plays an Essential Role in Viral Infection as an Uncoating Regulator. PLoS Pathog..

[27] Ahmad S.A.A., Palanisamy U.D., Tejo B.A., Chew M.F., Tham H.W., Hassan S.S. (2017). Geraniin extracted from the rind of Nephelium lappaceum binds to dengue virus type-2 envelope protein and inhibits early stage of virus replication. Virol. J..

[28] Spurgers K.B., Hurt C.R., Cohen J.W., Eccelston L.T., Lind C.M., Lingappa V.R., Glass P.J. (2013). Validation of a cell-based ELISA as a screening tool identifying anti-alphavirus small-molecule inhibitors. J. Virol. Methods.

