# Supplementary material for: Functional Insights into Silymarin as an Antiviral Agent against Enterovirus A71 (EV-A71)
Source: Int J Mol Sci. 2021 Aug 15;22(16):8757. doi: 10.3390/ijms22168757 (PMC8395941; doi:10.3390/ijms22168757)
Supplement: Supplementary file 1 [file ijms-22-08757-s001.zip › ijms-1230665-supplementary.pdf]

Supplementary figures

S. Figure 1:

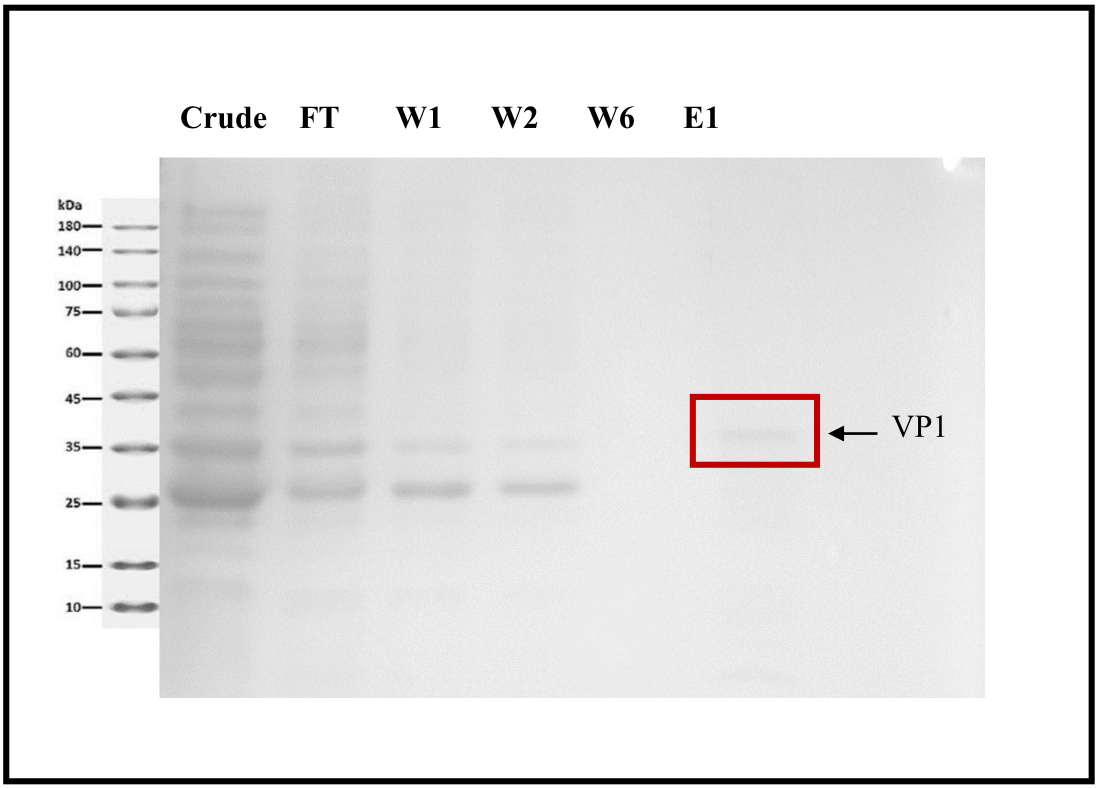

S. Figure 1: Expression of VP1 protein in *E.coli BL21*. Lanes: Crude: cell lysate induced with 0.1 mM IPTG; FT: flow-through, W1, W2 and W6: Washes 1, 2 and 6 were fractions eluted with wash buffer containing 10 mM imidazole and the final eluate (E1) was eluted from Ni-NTA column with 500 mM imidazole. A band at the 37 kDa indicated the purified recombinant VP1.

S. Figure 2:

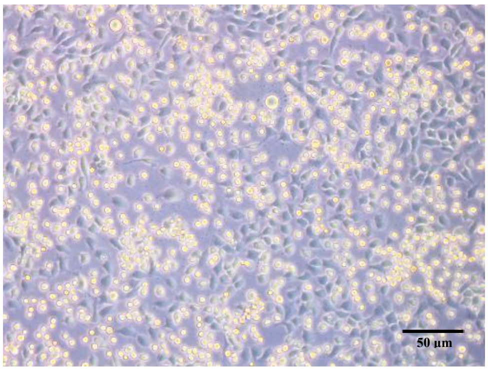

CPE caused by V1

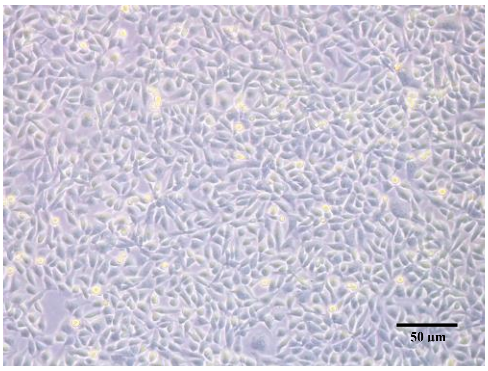

CPE caused by S1

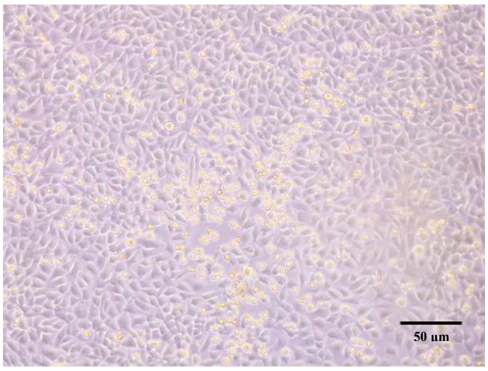

CPE caused by V9

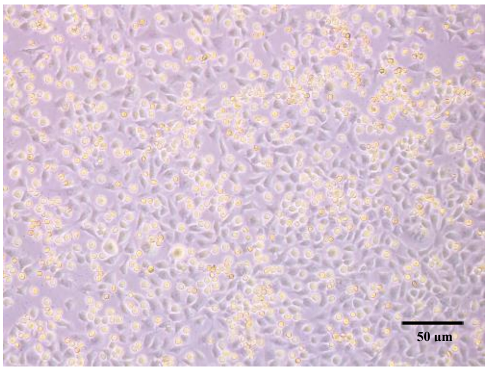

CPE caused by S9

S. Figure 2: Appearance of cytopathic effects (CPE) in Enterovirus 71 treated with silymarin over 9 continuous passages. The virus was passaged in RD cells in the presence of 100 µg/mL silymarin. Cytopathic effects (CPE) were observed and images were taken at 10X magnification. Wild-type virus was designated as V1, virus isolated after 9 passages in the absence of silymarin was designated as V9. Silymarin-treated virus after first passage was designated as S1 and virus isolated after 9 passages in the presence of silymarin was designated as S9.
